# Nutritional and Non-nutritional Composition of Human Milk Is Modulated by Maternal, Infant, and Methodological Factors

**DOI:** 10.3389/fnut.2020.576133

**Published:** 2020-09-16

**Authors:** Tinu Mary Samuel, Qianling Zhou, Francesca Giuffrida, Daniel Munblit, Valérie Verhasselt, Sagar K. Thakkar

**Affiliations:** ^1^Nestlé Research, Lausanne, Switzerland; ^2^Department of Paediatrics and Paediatric Infectious Diseases, Institute of Child's Health, Sechenov First Moscow State Medical University, Moscow, Russia; ^3^Inflammation, Repair and Development Section, National Heart and Lung Institute, Faculty of Medicine, Imperial College London, London, United Kingdom; ^4^University de Nice Sophia Antipolis, Nice, France; ^5^Nestlé Research, Singapore, Singapore

**Keywords:** human milk, lactation, maternal, infant, human milk sampling, standardization, human milk composition

## Abstract

Human milk (HM) is dynamic and shows a high inter- and intra-individual variability. To characterize HM with precision, it is necessary to understand the factors that modulate its composition. The objective of this narrative review is to summarize the maternal, infant and methodological factors that affect HM composition. We searched SCOPUS and PubMed databases for articles related to factors that are known to or could potentially influence HM composition and volume across lactation periods. Our comprehensive review encompasses various maternal-, infant-related, and methodological factors that modulate aspects of HM composition including macro- and micronutrients, vitamins and minerals, as well as volume. The most profound changes were observed in HM lipids and lipophiles. Evidence exists for many of the infant-related factors known to affect the nutritive and non-nutritive components of HM (e.g., birth weight, gestational age, infant age/stage of lactation). In contrast, less is known with respect to maternal factors; where there is either limited research or conflicting evidence (e.g., maternal lifestyle, obstetric history, medical conditions), except for the mother's diet, for which there is a relatively well-established understanding. Equally, although many of the methodological factors (e.g., HM sampling, handling and analytics) are known to impact HM composition, few studies have investigated this as a primary outcome, making it an important area of future research in HM. Here we propose a systematic capture of numerous maternal- and infant-related characteristics to facilitate associative comparisons of HM data within and across studies. Additionally, it would be prudent to standardize the methodological aspects known to affect HM composition in analytics, not only for HM lipids and lipophiles, but also for those nutrients whose variability is yet less well-understood. Defining the factors determining HM composition with accuracy will open perspectives for maternal intervention to optimize milk composition for specific needs of infants.

## Introduction

Breastfeeding i.e., human milk (HM^1^) feeding either from the infant's own mother, or wet nurses as in the past, was and continues to remain the feeding norm for new-born infants. Benefits of breastfeeding for both infants and mothers, in short- and long-term are well-documented (1–5). A recent publication indicates a protective effect of breastfeeding against childhood infections and malocclusion, increases in intelligence, and probable reductions in overweight and diabetes later in life (6). Mothers may also benefit from breastfeeding in that risk of breast, uterine and ovarian cancer is reduced, and post-partum weight loss is promoted (6). With multiple benefits of breastfeeding, it is no surprise that characterization of HM composition is of increasing interest in order to gain insights into the underlying factors that contribute to the benefits of breastmilk.

HM is a highly complex, and dynamic biological fluid rich in nutritive (e.g., lipids, proteins, carbohydrates, fatty acids, amino acids, minerals, vitamins, trace elements, etc.) and non-nutritive bioactive components (e.g., cells, Ig, cytokines, chemokines, hormones, growth factors, glycans, mucins, etc.). It provides protection against infection and inflammation, and contributes to immune maturation, organ development, microbiota colonization and overall infant health (7–9).

It is hypothesized that HM is tailor-made by each mother to meet the nutritional needs of her growing infant; plasticity of HM composition may be key to early infant growth and programming of health in later life. Studying HM composition is essential to not only understand the nutritional needs but also to correlate with other developmental outcomes of infants (e.g., neurodevelopment, immune development, and gut maturation). Understanding the associations between HM composition and developmental outcomes can also lead to strategies for modifying maternal nutrition in cases where needed and to adapt fortification strategies for preterm infants.

While there exist published reviews in which a selection of factors that influence the composition of HM is described, to our knowledge no review considers the numerous aspects that affect the composition and volume of HM. With our narrative review we aim to collate the most relevant factors that influence nutritional and non-nutritional composition of HM, namely both maternal- and infant-related factors. In addition, we highlight methodological aspects that affect the quantification of HM components in research settings, which in turn leads to a set of scientific recommendations for HM sampling, handling, and analytical characterization when embarking on research in this field. On the one hand, we attempt to be as comprehensive as possible. On the other, it would simply be too much for one paper to provide an in-depth review of all topics that modulate HM composition and volume. Therefore, the purpose here is to identify and focus on the most important factors including maternal diet, supplementation, galactagogues, maternal anthropometry, obstetric history, socio-demographic factors, medical conditions, as well as infant-related factors such as infant birth weight and sex. Temporal changes in HM volume and composition are also included. In addition, methodological factors affecting the quantification of HM components are assessed. Other factors are deprioritized but summarized and listed as further reading.

## Methodology

### Literature Search

We conducted a narrative literature review and search using SCOPUS and PubMed database with predefined keywords for all scientific literature related to factors that are known to or could potentially influence HM composition and volume across lactation.

An initial search was performed limited to titles, key words, and abstracts to assess the potential yield of the search strategy. Based on the results, search terms were modified (e.g., wild characters) or additional terms were included. A subsequent search included MeSH terms. The search terms were grouped into 2 different concepts, which were combined with Boolean term “AND.” Terms within each concept were combined with the Boolean term “OR.”

Concept 1: human milk, breast milk, mothers' milk, mothers' own milk, colostrum

Concept 2: maternal/mothers' diet, supplementation, anthropometry, smoking, alcohol consumption, coffee consumption, physical activity, breastfeeding frequency, parity, mode of delivery, cesarean section, age, socioeconomic status, psychology, drugs, galactagogues, infant sex/gender, gestational age at birth, small for gestational age, low birth weight, foremilk/midmilk/hindmilk, preterm, feeding time, type of expression, storage temperature/length, milk processing/pasteurization.

The search was limited to English language abstracts and articles only.

### Selection Criteria

We did not eliminate any kind of study design for initial screening as long as it met search criteria. Articles include original research, literature review and conference abstracts/papers/presentations that were published up to December 2018. After a final selection by reading the abstract of each article, 260 relevant papers were included.

## Results

Factors that impact HM composition and/or volume are summarized into the following two categories:

1. Maternal and infant characteristics affecting HM components
2. Methodological parameters affecting the quantification of HM components

The resulting information leads to a set of scientific recommendations for reproducible HM analysis in interventional as well as observational studies (see Methodological factors affecting the quantification of HM components in a research setting).

### Maternal Factors Affecting HM Volume and Composition

#### Maternal Diet

Maternal diet is an important factor that influences the volume and composition of HM. Some components have been studied extensively, while others require further research to draw strong conclusions. The results of our analysis are summarized in Table 1. Furthermore, a systematic analysis of the influence of maternal diet on HM composition, micro- and macronutrients, emphasized the relation of fatty acid intake, the intake of fat-soluble vitamins, vitamin B1, and vitamin C to their content in HM (10).

**Table 1:** Maternal factors affecting nutritive and non-nutritive components of human milk.

**Factors**	**Effect on nutritive or non-nutritive components of HM**	**Evidence**	**References**
Dietary or supplementary intake	Changes in maternal intake modify amino acids, (n-3) FA, iodine, selenium, thiamine, riboflavin, niacin, vitamin B6, vitamin B12, choline, vitamin C, vitamin A, provitamin A carotenoids, vitamin D, vitamin K	Evidence probable	(10–67)
	Changes in maternal intake do not modify calcium, magnesium, copper	Evidence probable	
	Changes in maternal intake do not modify HM volume, energy, macronutrients, phosphorus, potassium, sodium, chromium, cobalt, fluoride, iron, manganese, molybdenum, zinc, folate, non-provitamin A carotenoids, vitamin E	Inconsistent or lacking	
Anthropometry	The higher the BMI the higher the concentration of SFA, n-6/n-3 FA ratio, leptin in HM	Evidence probable in high-income countries Lacking in low-middle income countries	(68–82)
	The higher the BMI the higher the HM intake by the infant	Inconsistent or lacking	
Parity	Higher parity increases amounts of proteins, lipids, and improves FA profile	Inconsistent or lacking	(83–89)
	Multiparous women show increased concentrations of iron and Ig	Inconsistent or lacking	
Mode of delivery	Affects iodine, microbiome, IgA, or bacterial diversity	Inconsistent or lacking	(88, 90–93)
Age	Influences the content of lactose, lipids, protein, TN, NPN, Na, K, Ca, P, Cu, colostrum Ig	Lacking	(83, 84, 86, 87, 94, 95)
	Maternal age is negatively correlated with FA profile and Zn	Lacking	
Socioeconomic status	Socioeconomic status is associated with lipids, proteins, IgA, and FA profile	Inconsistent or lacking	(83, 95–98)
Medical conditions	Maternal GDM delays the initiation of lactation or reduces hormones	Inconsistent or lacking	(99–127)
	Maternal post-partum depression is associated with a shorter duration of lactation and perceived HM production	Inconsistent or lacking	
	Celiac disease reduces immune protective compounds (TGF-β1, sIgA) and bifidobacteria	Lacking	
	Allergies affects levels of interleukins, growth factors, beta-casomorphin, selective proteins, pro-inflammatory markers, cytokines	Inconsistent or lacking	
	Mastitis results in higher levels of minerals, MFG, and chlorides as well as higher catalase activity and increased concentration of IL-6	Lacking	
	Mastitis results in lower contents of lactose, lipid, protein, and casein	Lacking	
Use of galactagogues	Increase HM volume	Inconsistent or lacking	(63–66)

##### Effects of maternal diet on macronutrient content in HM

The macronutrient composition of HM is relatively constant across populations despite variations in the nutritional status of the mother (7). The effect of maternal energy intake on HM volume has shown conflicting results. Some studies showed that provision of additional calories (11, 128), energy restriction (12), or fasting (13) had no impact on HM volume, while others showed that a low calorie diet (1,200 kcal per day for 3 days) (14) or low energy intake (15) resulted in reduced HM production. In terms of macronutrient composition, an isocaloric high fat diet (16) and increased dietary consumption of proteins (17) and carbohydrates (18) demonstrated an increased HM fat concentrations at different stages. No significant differences in energy content of HM were found up to 12-weeks post-partum supplemented with a commercially available product, despite the higher energy intake (19). The same study revealed a greater volume of HM in mothers with a low mid upper arm circumference (<24.1 cm) following supplementation. Several reviews have highlighted the population differences in HM fatty acid composition and the influence of maternal diet on levels of PUFAs [including HM linoleic acid (LA), α-linolenic acid (ALA), and DHA] (20–22). A study provided supporting evidence that the quality of fatty acid intake during pregnancy and lactation is important (23). In this study the abundance of n-6 fatty acids (FA) and low n-3 FA, was reflected in the fatty acid composition of HM, in as such that DHA concentrations were significantly reduced particularly during lactation compared to pregnancy, while arachidonic acid (AA) did not follow the same development. On the contrary, women who consumed 2 portions of salmon per week from 20 week of pregnancy until delivery showed higher proportions of EPA (80%), DPA (30%), and DHA (90%) on day 5 post-partum (24). The impact of omega-3 supplementation during pregnancy and/or lactation on HM FA is well-studied: a systematic review found a positive relationship between the consumption of omega-3 sources and their concentration in HM, despite differences in the methods used, timing of supplementation, source of omega-3 source used, and the sample size (25). Supplementation with fish oil starting at 20 weeks of pregnancy until delivery not only increased the long-chain omega-3 FAs in HM during early lactation (up to 6 weeks post-partum), furthermore DHA levels at day 3, 6, weeks and 6 months were positively associated with infant DHA status at 1 year (26). Supplementing lactating women 4–6 weeks post-partum with either 200 mg or 400 mg DHA for 6 weeks with usual diets led to a 50 and 123% respective increase in HM DHA levels (27).

In terms of carbohydrate type, a randomized crossover study revealed that the consumption of a high-fructose corn syrup–sweetened beverage increased concentrations of fructose in HM, which stayed elevated for 5 h (28). No such effects were found for glucose or lactose. The consequences of habitual consumption of high-fructose sweetened beverages for early development need to be further investigated, but evidence exists that fructose in HM is positively associated with infant's body composition (29).

##### Effect of maternal mineral supplementation on mineral content of HM

Iodine supplementation (75–400 mg iodine/day) increased HM iodine concentrations (30–32), although a lower dose (75 or 150 μg/d) through either supplementation (31) or fortification (33) may be insufficient to ensure adequate iodine status in women or their infants. Equally, administering a single dose of 400 mg iodine as oral iodized oil to mothers may be an effective strategy to provide adequate iodine to their infants through HM for at least 6 months compared with direct supplementation to infants (30). The results of Mulrine et al. where iodine concentrations in HM were 1.3 and 1.7 times higher in women supplemented with 75 and 150 μg per day would also suggest a dose-response relationship (31). A systematic review of epidemiological and clinical data suggested that a high dose as well as daily iodine supplementation were effective in increasing iodine concentrations in HM (34). The same authors also concluded that iodine concentrations of around 150 μg/l during the first 6 months of lactation are enough to meet infants' needs. Selenium (Se) supplementation at a dose of 20 μg/day, approximating the quantity secreted into HM, (35) or 200 μg/day (36) increased HM selenium concentrations accompanied by an increase in infant Se intake. It is known that HM concentrations of minerals such as zinc and iron are not largely altered by deficiency or excesses in the maternal diet as these have well-regulated homeostatic processes (37). Zinc supplementation studies (10–50 mg/day) provided inconsistent findings, with some reporting an increase or prevention in decline in HM zinc concentration compared with placebo (38), while others did not find an effect of zinc supplementation (39, 40). Observational studies show reduced levels of iron in the HM of anemic mothers (41) and iron supplementation among women with low baseline levels of iron, was shown to increase HM iron concentrations (42). However, studies among healthy lactating women have failed to demonstrate an association between iron intake of the mother and HM iron concentrations (37, 43). There are physiological changes that happen during lactation, such as changes in calcium and bone metabolism, that provide sufficient calcium for HM production independent of maternal calcium supply in populations where calcium intakes are close to current recommendations (44). Furthermore, dietary nutrient consumption had no strong association with calcium concentrations in HM of three different ethnic groups in New Zealand (45). In this study, calcium intake of the mothers varied from 736 to 1,041 mg/day, but had no significant effect on the calcium concentration in HM.

##### Effect of maternal vitamin supplementation on vitamin content of HM

Maternal supplementation of B vitamins during lactation is known to rapidly increase their concentrations in HM as reviewed extensively Allen et al. (46). Evidence suggests that HM concentrations of thiamine, similar to those observed in well-nourished mothers, can be maintained by maternal thiamine intake of 2 mg/d (47). The concentrations of vitamin B6 in HM (ranging from 0.89 to 1.31 nmol/L) appear to be saturated at an intake of 2.5 mg/d of vitamin B6 (48, 49). Vitamin B12 supplementation (50 μg/d) was shown to maintain elevated concentrations of B12 in HM only during the supplementation period (6-week post-partum) (50) and the concentrations observed were much lower than those reported with a higher dose of 250 μg/d dose (97 vs. 235 pmol//L) (51). Choline supplementation of 750 mg/d increased HM concentrations of free choline, betaine, and phosphocholine (52), while a higher dose (930 mg/d) resulted in significantly higher concentrations of phosphocholine and glycerylphosphorylcholine (GPC), the main choline metabolites in HM as well as HM trimethylamine N-Oxide (TMAO), which is essential for maintaining osmotic balance and glycine concentrations (critical for production of glutathione that appear limiting in early development) (53). Similarly, higher lutein and zeaxanthin concentrations in HM have also been observed with lutein supplementation of 6 mg/d (140% higher) and 12 mg/d (250% higher) compared with the placebo (54). Further evidence comes from a study with Korean mothers, where a higher dietary intake of lutein was associated with higher lutein content in HM (55). In this study, the mean intake of lutein was 4.7 mg/day, with a range from 0.64 to 17.05 mg/day, while the mean lutein concentration in HM was 3.5 μg/dl, with a range from 0.5 to 31.3 μg/dl. Although no intervention studies have been reported so far, observational data suggest that the concentrations of biotin in the HM decreased over the course of lactation despite an intake of 57 mg biotin/d which is 63% higher than the adequate intake of 35 mg biotin/d, suggesting that a low intake of biotin may accelerate the decrease (56). There is strong evidence that retinol or beta carotenoid concentrations in colostrum (1–5 days) or mature (>14 days) HM are increased through supplementation of vitamin A during lactation either as retinyl palmitate (658–7,218 μg per day), as multivitamins containing β-carotenoids (providing 90 mg/d beta carotene) or as part of a diet enriched with vegetable oils like red palm oil (providing 90 mg/d beta carotene), soybean oil or sunflower oil (22). The same is true for a higher vitamin A consumption (range of 50–7,205 μg retinol activity equivalents), which led to higher, but non-significant, retinol concentrations (57). A strong positive association was demonstrated between maternal vitamin D intake during exclusive breastfeeding and infant serum 25-hydroxyvitamin D levels (58), although doses at the current recommended daily intake of vitamin D may not be sufficient for adequate transfer from mother to the breastfed infant (58) and supplementation during lactation with 4,000–6,400 IU/d of vitamin D may be needed to prevent vitamin D deficiency in the mother and her breastfed infant (59). The mode of supplementation needs to be taken into account, as a randomized controlled study demonstrated that a single bolus led to a higher production of vitamin D, compared with a daily dose (60), and levels remained elevated for at least 28 days. Among women with adequate folate status, HM folate secretion was shown to reach a maximum threshold and was not affected by the maternal folate status, except in clinically folate-deficient mothers (46). Maternal supplementation with alpha-tocopherol was shown to increase the vitamin E concentrations in colostrum, although the evidence is limited (61).

##### Galactagogues

Adequate lactation is fundamental for the (premature) infant's health, hence there is an increasing interest in galactagogues. Galactagogues are substances that have the potential to assist in various stages of milk production, such as initiation, continuation, or augmentation (62). Pharmaceutical agents, such as domperidone, or more recently herbs such as silymarin-phosphatidylserine and galega are widely used (63, 64). Many herbal concoctions have been proposed as galactagogues around the world. However, there is more anecdotal evidence rather than scientific rigor. There is a huge cultural as well as regional aspects on what is considered or promoted as a galactagogues. From many herbal galactagogues, fenugreek has most number of clinical trials reported in the literature and a recent network meta-analysis demonstrated its efficacy against placebo (65). Moreover, a study reported that a herbal tea containing stinging nettle, among other herbs, increased milk production in 36 mothers with premature infants by 80%, a significant increase when compared with the placebo and control group (66). Overall, the quality of galactagogue literature was found to be poor and the field would benefit from well-designed double blinded and randomized controlled studies.

#### Maternal Anthropometry

##### Influence of maternal weight/body composition on HM lipids and fatty acid profile

HM of overweight women is reported to contain higher amounts of SFAs, lower amounts of n-3 FAs, a lower ratio of PUFAs to SFAs and a higher ratio of n-6 to n-3 FAs, compared with normal weight women, even after adjusting for maternal diet (68). A study with 80 women reported a significant maternal age and body mass index (BMI) interaction with calorie and fat. BMI alone was significantly associated and protein content (69). Calorie and fat contents were found to be lower in overweight mothers in their 20s but higher in older, overweight mothers. The reason for this unique finding is not fully understood. The overall nutritional status of the mother and the stage of lactation may be important factors that deserve attention while studying the association between maternal BMI and HM macronutrient concentrations. To exemplify this, studies among well-nourished lactating women have failed to demonstrate any association between maternal fat stores and HM lipids (70), while those conducted among marginally nourished populations showed a positive relation both in early (<90 d) and late (≥90 d) lactation (71, 72).

##### Influence of maternal weight/body composition on volume of HM

Maternal body composition not only influences the composition of HM in terms of its macronutrient composition, but is also associated with the volume of HM transferred to infants when assessed using the “dose to the mother” deuterium dilution method (73). This was shown in infants from Kenya, where HM intake was positively correlated to maternal triceps and mid upper arm circumference during pregnancy (74). In contrast, among low-income Honduran women maternal anthropometric status was not found to be a significant predictor of HM volume or infant energy intake between 4 and 6 months of age after accounting for confounding factors such as birth weight and milk energy density. This highlights the ability of the infant to self-regulate intake in response to milk energy density and infant weight (75). Furthermore, studies reported positive associations between maternal BMI and HM leptin levels (76–79). One study found higher maternal %FM associated with higher leptin but not adiponectin concentrations (80). The same authors hypothesized that HM adiponectin levels are less likely to be impacted by maternal adiposity as the majority of HM adiponectin may be synthesized and controlled by the mammary gland. Others reported positive associations between HM leptin and adiponectin concentrations with infant weight gain (77, 78, 81) as well as HM adiponectin concentrations and fat mass in the infants up to 2 years (82).

#### Maternal Obstetric History

##### The influence of parity on HM composition

Parity has been found to be an independent factor influencing proteins and lipids content (83) and FA profile (84). However, findings are not consistent in the literature. Bachour et al. (83) reported an increased lipid concentration of HM associated with higher parity among Lebanese mothers. In contrast, among rural Gambian women, the amount of HM fed to the infant and the proportion of endogenous FAs in HM was markedly lower among those who had borne 10 or more children, implying an impairment of the ability to synthesize HM FAs *de novo* for high parity women (85). In terms of mineral content, increased amounts of iron were found in multiparous women (86). Islam et al. (87) did not find an association between parity and colostrum Ig concentration (including IgA, IgM, IgG, and peripheral immune cells) among women from Bangladesh, yet another study showed, increased IgA and IgM in colostrum of primiparous compared with multiparous Brazilian women (88). It is further hypothesized that a mother's breastfeeding of higher birth-order infants would be exposed to a wider array of organisms from their other children, and these could affect milk cytokine levels (89).

##### The influence of mode of delivery

Mode of delivery, including cesarean and vaginal delivery, was shown to affect protein content in colostrum (90), HM choline level (91), HM iodine concentration (92) and IgA concentration (88). Some data also suggested that vaginal delivery is associated with higher protein content in colostrum when compared with cesarean delivery (90). Ozarda et al. (91) found higher levels of choline in HM after cesarean vs. vaginal delivery. Iodine concentration in the transitional milk was found to be higher in women with cesarean section (349.9 μg/kg) in comparison with those with vaginal delivery (237.5 μg/kg, *p* < 0.001) (92). Higher IgA concentration was found in colostrum of women having undergone cesarean section rather than vaginal delivery, because of the occurrence of labor together with surgical stress (88). However, in a multi-center study, mode of delivery was not found to be a significant factor in IgA concentrations in HM (93). The effect of the mode of delivery on HM IgA concentration is suggested to be confirmed in larger cohorts (89). While we have started to observe the differences in HM composition between groups, further studies should focus on the implications of such differences on the health outcomes of the infants.

#### Maternal and Socio-Demographic Factors

##### Maternal age

The potential influence of maternal age on HM composition is still being debated. Early studies indicated that the lactose, fat, total nitrogen, protein nitrogen, non-protein nitrogen, sodium, potassium, calcium, and phosphorous concentrations differed little between adolescent and adult breastfeeding mothers (94). Likewise, maternal age was not related to colostrum Ig concentration (including IgA, IgM, IgG, and peripheral immune cells) (83, 87), milk lipids (83, 95), proteins (83), and copper contents (86). In contrast, Antonakou et al. reported maternal age as an independent factor, demonstrating a strong negative association between maternal age and MUFAs, including oleic acid proportions particular during the first months of lactation (84). Also, Silvestre et al. reported lower zinc contents in HM from older women when compared with HM from younger women (86).

##### Maternal socioeconomic status

There is no consistent evidence about the influence of maternal socioeconomic status on HM lipid contents. Rocquelin et al. did not find an association between socioeconomic status, e.g., mothers' education/occupation and HM lipid content among Congolese mothers of 5 month old infants (95). Bachour et al. found a notable but not statistically significant association between maternal residential area and milk lipids, proteins and IgA levels (83). In contrast, Al-Tamer and Mahmood demonstrated an effect of maternal socioeconomic status of lactating mothers in Iraq on HM lipid content, TG and FA composition, especially the proportions of long-chain omega-3 FAs decreased with decreasing socioeconomic status (96). Similarly, a study in low-income Indian women demonstrated an association of socioeconomic factors, particularly maternal education, with HM composition in as such that a higher education resulted in lower concentrations of SFAs and PUFAs (97). In Chinese women a higher education was positively associated with concentrations of carotenoids and tocopherol in HM (98).

##### Geographical location

There is consistent evidence in the literature that HM FA composition varied according to geographical location, and variation was frequently attributed to the differences in maternal diet (129–133). For example, Chulei et al. reported different concentrations of DHA and AA: DHA ratio in HM in five distinct geographical regions (pastoral, rural, urban 1, urban 2, and marine) of People's Republic of China (130). Roy et al. revealed differences in HM n-6 and n-3 PUFAs between urban and suburban mothers in Bengali, India, and contributed the rural and urban differences to the mothers' alimentary habits (134). A study comparing the levels of omega-3 and omega-6 PUFAs in HM of Swedish and Chinese women demonstrated a more favorable balance of those FAs in Swedish women (135). In addition to lipid content, mineral composition of HM, namely phosphorous was found to vary among three different regions among 444 Chinese lactating women (92). Oligosaccharide profiles in colostrum varied between Italian and Burkinabe women, in as such that in contrast to Italian women, the Burkinabe women had higher concentrations of 2 fucosyllactose and lower concentrations of lacto-N-fucopentaose in colostrum (136). The relevance of these findings is not clear. Like FAs, growth factors vary according to geographical location. For example, 228 breastfeeding mothers from 3 regions in People's Republic of China revealed significant differences in the content of epidermal growth factor (EGF) and TGF-α content in HM by region (137). Differences in diet may explain some of the findings as the concentration of EGF in HM significantly decreased with increasing intake of proteins, total energy, vegetables, fruits, soy products and dairy foods, while the TGF-α content in HM significantly increased with increasing intake of carbohydrates and dairy products and decreased with increasing intake of proteins and meat. Overall, we hypothesize that geography is a proxy factor for underlying factors that drives the differences in HM composition. The major factor that remains common to a cohort in geography such as diet or life style are usually the underlying causes of demonstrated differences in HM composition.

##### The influence of maternal exposure to toxins on levels of toxins in HM

Human milk of mothers with different childhood exposures differs significantly. It might be possible that exposure of the mother during her own infancy primes her intestinal immune cell development in as such that when immune cells migrate from the maternal intestine to the mammary gland, results in altered HM composition. It was reported that women who were breastfed during infancy and grew up on the Baltic coast of Sweden had high levels of polychlorinated biphenyl (PCBs) and polychlorinated dibenzo-p-dioxins (PCDDs)/polychlorinated dibenzo-furans (PCDFs) in HM. Exposure early in life to HM and consumption of contaminated fish from the Baltic sea were the reasons for high toxic level of PCBs and PCDDs in their HM (138). Also, toxic metal contamination in HM is of concern (139). The authors found, for example, arsenic in 64% of HM samples in Lebanese women and identified an association with cereal and fish intake. In general, inter-country studies reported varying levels of perfluorinated carboxylic acids in HM samples from Japanese, Chinese and Korean mothers, with the highest levels in Japanese mothers (140). More specifically, a strong positive association of perfluoroalkyl substances in HM with maternal age, BMI and parity was reported from an analysis of HM from 128 Korean mothers (141). The same authors reported the consumption of snack, milk, and eating-out frequency to be associated with increased levels of perfluoroalkyl substances.

#### Maternal Medical Conditions

##### Effects of gestational diabetes mellitus (GDM) on composition of HM

Maternal prenatal metabolic profile influences the composition of HM (99). It was shown that mothers with diabetes experienced lactogenesis II later than healthy mothers (100). A relatively small study showed that levels of ghrelin, an appetite-stimulating hormone, were lower in HM of early lactation of mothers with GDM as compared with mothers without GDM, but that ghrelin levels were restored to normal in mature HM (101). These data need to be confirmed in a larger group of women. Moreover, mothers with a higher pre-pregnancy BMI combined with GDM or lower insulin sensitivity had higher insulin concentrations in mature HM compared with normal glycaemic mothers (102). Galipeau et al. found that the presence of gestational diabetes increased the risk of an elevated HM sodium level at 48 h after delivery (127). However, Klein et al. did not find any significant differences in HM free AAs content between GDM and healthy women, regardless of it being colostrum or mature HM (103).

##### Influence of celiac disease on composition of HM

In comparison to healthy mothers, HM of mothers with celiac disease was found to have a reduced abundance of immune protective compounds (TGF-β1 and sIgA) and bifidobacteria (104). On the other hand, HM of 42 mothers with celiac disease who followed a gluten-free diet showed no difference in anti-gliadin antibodies compared with HM from 41 mothers with a normal, gluten-containing diet (105). This suggests that rather than diet, the immunological memory defines the presence of these antibodies in HM.

##### Influence of maternal atopy/allergy on composition of HM

The association between maternal atopy/allergy and HM composition is not consistent in the literature. Higher IL-4, IL-5, IL-13 (106), and beta-casomorphin-5 (107) in colostrum, and higher levels of IL-4 (108), lower levels of TGF-β1 (109, 110), TGF-β2 (111), IL-10 (108) and proteome (including protease inhibitors and apolipoproteins) (112) in HM were found in women with history of allergic disease compared with non-allergic women. Higher levels of sCD14 were found in mothers with a positive vs. negative allergic history (113). On the contrary, Marek et al. (109) did not find any difference in IL-4 and IL-10 levels in HM between allergic and non-allergic mothers. Likewise, no differences were reported in pro-inflammatory markers and cytokine concentrations (111, 114, 115) and FA composition (111) in HM between allergic and non-allergic mothers. A study among Danish mothers with atopic dermatitis, mothers with other types of atopy, and non-atopic mothers reported that the HM FA composition was not affected (116). In contrast, women with a combination of eczema and respiratory allergy had lower HM levels of several PUFAs (AA, EPA, DHA, and DPA), and had a lower ratio of long-chain n-3 PUFAs/n-6 PUFAs, even though the fish consumption was not different between groups (117). Their PUFA levels differed not only from that of healthy women, but also from that of women with only respiratory allergy, who had a FA pattern like that of healthy women.

##### Mastitis

Early evidence indicated that HM of mothers with mastitis showed a higher content of minerals, chlorides, and catalase activity, and lower levels of lactose, fat, total proteins, and total casein fractions, in comparison with HM from healthy women (118). This observation is likely because mastitis inflammation can produce pro-inflammatory cytokines and damage the milk fat globule (MFG). Mizuno et al. found that HM of mothers with mastitis contained larger MFG and higher IL-6 levels in the diseased breast than HM from the healthy breast (119). The same authors reported that this difference was larger if accompanied by systemic symptoms of mastitis (fever/malaise). In a separate study, Buescher and Hair reported that HM of mothers with mastitis had the same anti-inflammatory components and characteristics of HM from healthy mothers; although elevation in selected components/activities was observed (TNFα, soluble TNF receptor II, and IL-1RA and bioactivities that cause shedding of soluble TNF receptor I from human polymorphonuclear neutrophils) (120). These components may help protect the nursing infant from developing clinical illness due to intake of HM from mothers with mastitis.

##### Post-partum depression

About 85% of women experience a so called “post-partum blues,” which is transitional and lasts ~2–3 weeks after birth (121). The “blues” does not resolve in some women, and about 10–20% of new mothers experience post-partum depression (122). Post-partum depression is frequently found to be related with a shorter breastfeeding duration (123, 124). Currently, there are no randomized controlled trials to demonstrated causality. A population-based prospective cohort study showed that maternal depression was associated with maternal perceptions of insufficiency of HM but not actual milk quantity (125). This finding was supported in lactating mothers of preterm and term infants, where the perceived mood states had no apparent effect on HM volume (126).

#### Other Maternal Factors Influencing HM Composition and Volume

For the sake of greater focus on the most relevant factors influencing HM composition and volume, this chapter lists but not discuss in detail other topics that could be considered for planning and conducting research in this area but are not discussed in detail. Instead, further reading is proposed (see Table 2). Indeed, many factors have significant impact on human milk composition, however, how those differential composition impacts infant outcomes should be a topic of future studies.

**Table 2:** Further reading for factors with potential impact on human milk.

**Factors potentially modulating HM composition and volume**	**Suggested reading**
Use of medication	(142)
Hormones	(143)
Stress	(144)
Microbiome	(145–148)
Ethnic groups	(45, 149)
Infant suckling time	(150, 151)
Smoking	(83, 152–155)
Alcohol consumption	(156–158)
Coffee consumption	(159–162)
Physical activity	(163–171)
Breastfeeding frequency	(127, 150, 151, 172)
Immunological composition	(89, 173)
Drug use	(142, 159)
Synbiotics	(67)

### Infant-Related Factors Affecting HM Volume and Composition

The main factors have been summarized in Table 3.

**Table 3:** Infant-related factors affecting nutritive and non-nutritive components of human milk.

**Factors**	**Effect on nutritive or non-nutritive components of HM**	**Evidence**	**References**
Birth weight	Higher birth weight increases HM intake	Evidence probable	(174–176)
Gestational age at birth	Preterm birth increases true protein in colostrum	Evidence probable	(177–184)
	Preterm birth decreases lactose in colostrum	Evidence probable	
	Preterm birth decreases minerals, Ig, growth factors	Lacking	
Sex	Male infants consume higher amounts of HM than female infants	Evidence probable	(185–188)
	Energy and lipid content of HM is higher after giving birth to male infants	Inconsistent or lacking	
Age (stage of lactation)	Advanced stage of lactation increases HM intake, as well as energy, total lipids and HMO (3 FL) content	Evidence probable	(4, 7, 98, 137, 186, 189–213)
	Advanced stage of lactation has no major impact on lactose and some HMOs (e.g., 3 SL)	Inconsistent or lacking	
	Advanced stage of lactation decreases total and major proteins, as well as some HMOs (e.g., 2 FL), immune factors, whey/casein, vitamins and zinc	Evidence probable	
Feeding session	Hindmilk has higher energy and total lipids compared with foremilk	Evidence probable	(195, 196)
Feeding—time of the day	Mid-day feeding demonstrates higher energy and total lipids	Lacking	(214, 215)
Variations between breasts	Milk output from the right breast is greater than from the left breast	Inconsistent	(195, 196, 216–224)
	Energy content in HM from the left breast is higher than from the right breast	Inconsistent or lacking	
Circadian variability	Lipids and lipolytic enzymes in HM peak at mid-day	Evidence probable	(150, 214, 215, 225–239)

#### Infant Birth Weight

A positive relationship has been suggested between birth weight and HM intake attributed mainly to increased sucking strength, frequency or feeding duration (70). In the DARLING study, weight at 3 months and total time nursing were positively associated with HM intake at 3 months, while no maternal variable (such as age, parity or anthropometric indices) was significantly correlated with intake, volume extracted, or residual milk volume, indicating that infant demand was the main determinant of lactation performance (174). In contrast, no differences were observed in absolute and relative HM transfer volumes and HM zinc concentrations of small for gestational age vs. appropriate for gestational age infants born to low-income, marginally nourished Bangladeshi mothers (175).

#### Infant Gestational Age at Birth and Term vs. Preterm Delivery

A meta-analysis of 41 studies indicated significantly higher concentrations of “true protein,” excluding the non-protein nitrogen, in the first few days post-partum. Lower lactose concentrations (in the first 3 days and some later time points) were found in preterm compared with term HM, although no differences were observed for fat and energy content (177). Compared with term HM, preterm HM was shown to have a highly variable human milk oligosaccharides (HMO) content and was richer in glycosaminoglycans. The latter allows HMOs to competitively bind to pathogens and prevent them from adhering to the enterocytes (178). With respect to mineral content, preterm compared with term HM is reported to have higher concentrations of copper and zinc that decline across lactation and lower concentrations of calcium that remains constant over lactation (178). HM from preterm mothers was shown to have higher levels of IgA, IL-6, EGF, TGF-β1, and TGF-β2 in colostrum compared with term HM (179). Ozgurtas et al. found higher levels of vascular endothelial growth factor, basic fibroblast growth factor (b-FGF) and insulin-like growth factor-1 (IGF-1) in HM from the mothers of preterm infants (180). Similarly, preterm delivery also affects hormone concentrations in HM. In a study with 40 lean mothers, those of the preterm infants showed higher concentrations of obestatin and higher expressions of ghrelin mRNA in mammary epithelial cells, however the latter did not result in higher ghrelin concentration in HM (181). It may be speculated that the synthesis of these peptides and their transport from maternal circulation could contribute to the finding that the transcript level of the gene does not correspond to the peptide level in HM. While the HM of mothers of preterm babies seemed to have a higher concentration of immunological markers, there is conflicting evidence on the ability of gestation age to influence HM PUFA composition. One study showed lower levels of EPA and DHA in HM from mothers of preterm infants (182), while others did not find any association (183). Limited data showed that gestation age may influence HM minerals composition. For example, Ustundag et al. reported lower zinc levels in HM from mothers of premature infants with a similar trend seen in colostrum copper levels (184).

#### Sex of the Infant

The sex of the infant may also be one of the determinants of HM volume and composition of HM, for example, the HM of mothers with male infants had 25% greater milk energy content than mothers delivering female infants (185). Furthermore, at 4 months of age, HM for male infants compared with females were higher in energy and lipid content by 24 and 39%, respectively (186). The same study revealed lipids such as linoleic acid, phospholipids and gangliosides were increased in HM of male infants in later lactation, potentially based on the higher energy requirement of a male infant (186). Interestingly, in resource poor setting, HM for female infants was richer in lipids, while in a resource sufficient setting, the milk for male infants was richer suggesting that infant sex and maternal socioeconomic status showed an interaction and should be studied further (187). HM intake, assessed using stable isotope methodology, was shown to be higher in males than in females over the first 12 months of lactation, likely related to the fact that males have greater lean mass than females during infancy (188).

### Temporal Changes in HM Volume and Composition

#### Changes in HM Volume Across Lactation

HM is a dynamic fluid that changes its composition and volume depending on the stage of lactation and adapts to the evolving nutritional requirements of the infant (4, 189–192). Stable isotope studies in infants aged 0–24 months have shown that the volume of HM intake increased over the first 3–4 months and remained above 0.80 kg/d until 6–7 months in order to meet the increasing energy requirements of the infant (188).

Branched-chain FAs may reduce the incidence of necrotizing enterocolitis, but evidence is limited. One study found branched-chain FAs decreased with increased lactation (193). Most growth factors (EGF, IGF, and TGF) significantly decrease over the course of lactation from colostrum, over transitional and mature milk, while TGF-α content in HM revealed a significantly increased trend over the course of lactation (137). The time of lactation had an influence on the concentration of oligosaccharides, on 4 out of 22 oligosaccharides measured. (194). However, published data are hampered by large variation and results need to be interpreted with caution.

#### Changes Observed Within Feed and Between Breasts

The energy content of HM is not constant and changes within a feeding (195). Significantly higher energy content has been observed in hind milk than in foremilk since the lipid content increases markedly with emptying of the breast. The fat content of hind milk is reported to being approximately two- to three-fold that of foremilk and included 25–35 kcal/100 mL more energy on average than foremilk (195). Milk volume differences between left and right breasts have also been a topic of study. Reports indicated that milk output from the right breast was usually greater than the left breast (216–219, 240). However, the opposite has also been reported where the left breast showed a greater milk output in pump-dependent mothers of non-nursing preterm infants (220). Perhaps studying physiological implications of left cradling bias that occurs in 70–85% of the women may help shed light on the observed between breast variability (221). When milk composition from the left and right breasts was assessed over 12 months in fore and hind milk the content of fat was found to vary slightly between breasts, with a mean CV of 47.6 (SE 2.1) % (*n* 76) and 46.7 (SE 1.7) % (*n* 76) for left and right breasts (196). These variations resulted in differences in the amount of fat delivered to the infant over 24 h. This indicated that a rigorous sampling routine should take into account all levels of variation in order to accurately determine infant HM intake (196). Different levels of sodium, chloride, glucose, lipid, and zinc were reported in the HM of breasts but differences were either transient and/or random (222). In a different study the levels of sodium, the levels have shown a difference, potentially due to underlying mastitis localized to only one breast (223).

#### Changes Due to Circadian Variability

Diurnal variation in the lipid content of HM was first studied more than a century ago (225). Later it was shown that diurnal variations were unrelated to the degree of emptying of the breast in the previous feeding and to maternal meal times (226). Since then it has been a topic of interest for many teams of scientists in normal term (150, 227–229, 241) as well as preterm infants (230). It is noteworthy that not only lipids in HM but lipolytic enzymes of HM show a circadian rhythm and peak at mid-day, which has been shown for serum-stimulated lipase and lipoprotein lipase but not bile salt-stimulated lipase (214, 215). In a study with 6 women, HM volume, along with total lipids were found to be significantly higher between 08:00–12:00 and 16:00–20:00 (231). The same team suggested that if 24-h sampling was not possible, single samplings at 12h00, 20h00, and 24h00 may be most representative for lipid yield and may predict the value within 97–124%. Since lipids provide more than 50% of total energy in HM, it is not surprising that energy content of HM was positively related to fat content (232). Though lipids have been studied the most, milk minerals also have received some attention. However, the evidence was not very conclusive and did not demonstrate a cyclic change at a different stage of lactation (233–237). Endocrine factors and melatonin are hypothesized to improve nocturnal sleep and reduce infantile colic (238). Indeed, it has been shown that melatonin is found at significantly higher concentrations in night time HM (7.3 pg/mL) as opposed to day time HM (1.5 pg/mL) (239).

#### Longitudinal Changes Across Lactation

Among all the factors covered in this paper, longitudinal changes observed across progressing stages of lactation have been studied the most. Key differences include the presence of a relatively high abundance of immune related factors in early compared with later stages of lactation (7). Additionally, a higher concentration of total proteins has been observed in colostrum and transitional compared with mature milk (190). In contrast, lipids are present at higher concentration in mature milk (186, 196). In next section we review the different aspects of changes across lactation as reported in the literature.

##### Changes in the macronutrient composition of HM

The energy content of HM showed an initial decrease followed by an increase over the course of lactation, which was directly related to the HM lipid content (186, 196). With respect to carbohydrates, several studies showed that lactose concentrations remained constant throughout lactation (186, 195, 197), however, there is also some evidence to the contrary, indicating an increase in HM lactose from 56 to 69 g/l over the first 4 months of lactation (198). The other significant carbohydrates found in HM are oligosaccharides, the concentrations of most of which, not all, have been shown to decline during the course of lactation although HM concentrations of 3-FL either increased throughout lactation or remained at relatively constant concentrations starting at 1 month onwards (199, 200). The colostrum to milk transition was also associated with significant changes in concentrations of free sugars and polyols (increased in concentrations of lactose and glucose, decreased in concentrations of Myo-inositol and glycerol) over the first 3 days after birth and thereafter reaching a steady state (201).

Colostrum contains very high concentrations of protein (ranging from 20 to 30 g/L), which decreases significantly thereafter, and reaches 7–8 g/L after 6 months of breastfeeding (202). This observation coincides with lower protein intake and lower growth rate of breastfed infants in the latter half of the first year of lactation (203). A systematic review reported on the reduction in HM protein concentration (g/L) from about 25 in colostrum (age 1–5 days) to 17 in transitional milk (age 6–14 days) and then to 13 (8–21) in mature milk (at age of >14 days and <6 weeks) (204). The protein profile of the HM also evolves over lactation. At the beginning of lactation the whey: casein ratio was around 90:10, reduced to 70:30 in transition milk, then to 60:40 in mature milk and ultimately reached 50:50 ratio in mature milk after 6 months of lactation (205, 206). The concentration of lactoferrin in HM (second most abundant whey protein in HM known for its antimicrobial activities) was high during early lactation (<28 days lactation), decreased by almost 50% in the first 5 days of lactation, and thereafter remained relatively stable (207). The total nitrogen and total AAs in HM decreased in the first 2months of lactation and thereafter remained relatively unchanged, while the free AAs (glutamic acid and glutamine) increased over lactation, peaked around 3–6 months, and thereafter decreased (208).

The most variable macronutrient in the HM is fat, which is dependent on a number of factors and includes the fat content of colostrum; it is usually very low (1–2%) but was shown to increase rapidly during the first week of lactation (204). The mean lipid content of HM (g/L) increased from 22 in colostrum (age 1–5 days), to 30 in transitional milk (age 6–14 days), and 38 in mature milk (age >14 days and <6 weeks) (204). There were also stage-based changes reported in the ganglioside content of HM with some decreasing and others increasing over the time (209).

##### Changes in micronutrient composition of HM

The concentrations of antioxidant vitamins such as A and E are known to be lower in mature milk compared with colostrum and decrease over the course of lactation, while the total antioxidant status of mature milk was higher than colostrum indicating an increase in total antioxidant properties (210). Non-provitamin A carotenoids (lutein and lycopene) decreased while there was no significant change in the provitamin A carotenoids (α- and β-cryptoxanthin) with advancing lactation stage (211). Over the course of lactation (up to 240 days) significant changes of carotenoids and tocopherol were reported in a population of 540 Chinese women (98). In this population, the concentrations of lutein, zeaxanthin, and α-tocopherol were higher the first 4 days, decreased until 12 days post-partum to remain stable afterwards. Zeaxanthin and γ-tocopherol remained stable over time.

Zinc concentrations in HM decline dramatically over the first 3–5 months of lactation and even an increase in HM volume during the early weeks post-partum is not sufficient to overcome this decline (212). A marked decrease in HM cobalamin was also observed at 4 months and this has been associated with an accompanying decrease in plasma cobalamin and holotranscobalamin in infants, indicating an impaired cobalamin status (213).

### Methodological Factors Affecting the Quantification of HM Components in a Research Setting

The main factors identified have been summarized in Table 4.

**Table 4:** Methodological factors affecting nutritive and non-nutritive components of human milk.

**Factors**	**Effect on nutritive or non-nutritive components of HM**	**Evidence**	**References**
Human milk expression	Hand expression compared with pump expression shows lower daily milk supply	Inconsistent or lacking	(242–247)
	Hand expression compared with pump expression results in higher sodium, potassium, proteins, total lipids	Inconsistent	
Storage temperature	Higher temperatures (−20°C) reduce total lipids	Inconsistent or lacking	(248–255)
Storage length	Longer storage durations reduce vitamin C concentrations	Inconsistent or lacking	(248, 252–255)
	Longer storage durations do not affect tocopherol concentrations	Evidence probable	
	Longer storage durations (12 months) reduce concentrations of IgA, IL-8, and TGF-β1	Inconsistent or lacking	
Freeze-thaw cycles	Multiple cycles reduce total lipids	Evidence probable	(250, 251, 256, 257)
	Multiple cycles reduce carbohydrate concentrations	Lacking	
	Multiple cycles increase lipolytic products (free FA, monoacylglycerol)	Evidence probable	
Choice of analytical method	Determination of combustion in a bomb calorimeter accurately quantifies macronutrients and total energy	Evidence probable	(177, 205, 258–273)
	The Kjeldahl method or micro-Kjeldahl analysis measure true protein with high precision	Evidence probable	
	High throughput spectroscopy measures protein accurately	Inconsistent	
	The Folch, Bligh and Dyer and Röse-Gottlieb methods accurately measure lipid content	Evidence probable	
	Spectroscopic methods determine total lipids accurately	Inconsistent	

#### Sample Collection—Hand Expression vs. Electric Pump Expression

It is well-known that there are multiple ways of expressing milk from mothers' breasts, be it for feeding to the new-born, for donation or for research purposes. The methods may include but are not limited to, hand expression, manual pump and electric pump (242). In a randomized trial of mothers delivering very low birth weight infants, hand expression produced significantly lower cumulative daily milk volume in comparison with electric pump expression (243). Sodium concentration was shown to be significantly affected by method of pumping. Higher sodium content was found in hand expressed milk as opposed to electric pump-expressed milk (244). In a Cochrane review of methods for HM expression, sodium, potassium, protein, and fat constituents of HM were reported to differ between methods. However, no consistent effect was found related to prolactin change or effect on oxytocin release with pump type or method (242). A randomized trial indicated that HM expressed manually contained higher fat than that expressed electrically (245). Morton et al. (246) indicated that a combination of manual technique and electric pumping resulted in high levels of fat-rich, calorie-dense milk, unrelated to production differences. An experiment from 57 lactating women showed that macronutrient content (fat, carbohydrate, protein, and energy contents) of mid expression HM is unaffected by maternal handedness, breast size or breast side dominance (247, 274).

#### Post-collection Handling of HM Samples

Utmost care must be observed with HM samples immediately after the milk is expressed from the mothers' breast into the collection container. Collection tubes may need to be acid washed prior to collection if the intention is to measure minerals. Similarly, maintenance of a cold chain until laboratory analyses, ensuring optimal storage temperature and duration, limiting freeze-thaw cycles, amount of oxygen exposures etc. comes into the scope of post-collection handling variability. It is our general observation that not many systematic studies exist that assess and guide the future HM research scientist, but few observational as well as data supported statements are mentioned in this section.

##### Storage temper and duration

Lipids, more than carbohydrates or proteins in HM, are sensitive to storage over long periods. Chang and colleagues demonstrated that even storage at −20°C for 2 days leads to ~9% reduction in HM total lipid content (248). It is likely that bile salt-stimulated lipases present in HM may still maintain some activity at cooler temperatures and lead to enzymatic hydrolysis of lipids (249). Indeed this was confirmed in a separate study where storage at −20°C was also done with pre-heating milk samples at 80°C (90 sec) to destabilize the lipolytic enzyme (250). Therefore, if possible, samples should be stored at the minimum possible temperature, preferably at −80°C, from the day of collection. However, others have argued that freezing at −80°C reduced the energy content of both fat and carbohydrates and suggested HM should be stored at −20°C (251). Furthermore, even vitamin C was reduced significantly over time in cold storage but not tocopherols and total FAs (252, 253). Bacterial composition (quantitative or qualitative) of HM was stable with cold storage at −20°C for 6 weeks (254). Long-term storage of HM may affect immunological composition. According to Ramirez-Santana et al. freezing storage of colostrum at −20 and −80°C for a 12-month period produced a decrease in the concentrations of IgA, IL-8, and TGF-β1 (255).

##### Freeze-thaw cycles

Freeze (−20°C) and thaw (5°C) cycles have been shown to activate lipolysis and increase the presence of lipolytic product in HM, such as free FAs, monoacylglycerols and diacylglycerols (250, 256). Structural effects such as increasing size of milk fat globules due to freezing have also been observed (257). Carbohydrate content was significantly reduced in thawed HM samples when compared with fresh samples (251).

#### Analytical Procedures

The choice of analytical methods to measure the nutrient of interest in HM is important, especially in comparative and longitudinal studies, where differences among populations and lactation stages could be due to biological variation or may reflect methodological biases and errors. Several factors influence the analysis of HM and in the following paragraph, we will discuss the most relevant ones. This is not intended to be a review on methodologies for determination of nutrients in HM, but to demonstrate the consequence of using unsuitable methodologies.

##### Sample preparation

Using the correct preparation and storage of samples are important elements of maintaining the integrity of HM during storage, as previously mentioned. In our experience, maintaining sample homogeneity is an issue both when frozen samples are thawed at room temperature or in a 37°C water bath. Often, probe sonication should be used if the measure is based on optical analyses, such as the commonly used mid infrared spectroscopy. HM must be immersed in warm water (30–40°C) and gently mixed, prior to analysis, in order to rapidly thaw and minimize phase separation. Homogenization is an important step in preparing milk samples for total fat determination by infrared (IR) spectroscopy techniques, as it decreases the variability in fat globule size and the light-scattering effect of larger globules, improving the accuracy of the measurement (258).

##### Selectivity/Specificity and accuracy

When quantitative data are required, the analytical methods chosen must demonstrate enough selectivity and accuracy. Selectivity is the ability of the method to distinguish between the analyte and other substances present in the matrix. In absence of selectivity, accurate values cannot be obtained. The accuracy expresses the closeness of a result to an accepted reference value and it is normally studied as two components: trueness, which represents the closeness of agreement between the average value obtained from a series of results and a reference value and precision, which is the closeness of agreement between independent test results obtained under stipulated conditions (ISO 5725) (259). In the following paragraphs, several methodologies are discussed with respect to selectivity and accuracy.

##### Energy

Two approaches have been used to measure energy in HM: direct energy quantification by combusting in a bomb calorimeter and calculated energy using Nichols et al. (260) energy multiplication factors for protein, fat and carbohydrate. Lack of accuracy has been shown by Gidrewicz et al. (177) for the measurement of energy when calculated using values for the energy contributions from fat, protein and carbohydrate rather when measured by bomb calorimetry. Differences as high as 13%, which likely represent clinically important differences, were reported (177). Since HM contains a large amount (~12%) of non-digestible oligosaccharides, they will contribute a significant amount of energy when using bomb calorimetry for energy measurement, but since they are essentially non-digestible, they should contribute significantly less energy to the infant. Overall, the choice of analytical methods can determine outcome and interpretation of HM analyses. Therefore, adoption of standard methods when possible and/or rigorous assessment of alternative methods is needed. To assure equivalence of two analytical methods, we recommend evaluating the accuracy of the alternative method and to check the presence of systematic and/or proportional bias by performing analysis of differences and a regression analysis, respectively.

##### Proteins

Amino acid analysis is the most accurate method for determining true protein content (205). The Kjeldahl method, based on complete combustion of the sample, measures total nitrogen (TN) and it is the most accurate indirect determination of total protein content in HM (261). The true protein content can also be estimated by the chemical determination of non-protein nitrogen (NPN) (262), and subsequent subtraction from TN. Unfortunately, AA analysis is costly and time consuming and the Kjeldahl method requires large amounts of sample. Alternatively micro-Kjeldahl (263) analyses can be used with high precision to determine true protein because it has been shown to correlate well with AA analysis (264) if corrections are made for NPN. High throughput approaches requiring low sample amounts such as spectroscopy and colorimetric methods have been investigated (258, 262, 265–268). Absence of accuracy was shown (258, 266) when comparing near- and mid IR spectroscopy equipment for determination of total protein against chemical reference methods. Casadio et al. (265) compared Miris HMA vs. Bradford for total protein determination and reported overestimation of protein content. On the other hand, Silvestre et al. (267) reported underestimation of protein content when Miris HM analysis was compared vs. Bradford for total protein determination. This observation illustrates that spectroscopy methods can vary in accuracy depending on the calibration of the instrument and reference method; hence the appropriate methods should be selected with care. Keller and Neville (262) compared micro-Kjeldahl to different spectrophotometric methods to determine proteins in HM: Biuret assay, Lowry-Peterson assay, Bio-Rad Coomassie Blue assay and Pierce Bicinchoninic acid assay (BCA). The Pierce BCA assay demonstrated better accuracy, and was recommended for total protein analysis in HM. Lonnerdal et al. (268) showed the BCA method consistently overestimated Kjeldahl protein by 30%. However, parameters such as incubation time and temperature highly influenced the accuracy of the BCA method (269).

##### Lipid

In order to accurately quantify HM lipid, the first step is disruption of the biological membrane enveloping triacylglycerol core, e.g., ultrasound treatment, followed by lipid separation from milk protein by the addition of organic solvent [e.g., methanol (270, 271)] or acids or bases [NH_3_ (272)] which triggers protein precipitation. At this point, lipid can be extracted from the matrix by using organic solvents. Since most of the lipid in HM is represented by triacylglycerol (98%), solvents like diethyl ether and petroleum benzene (272) can be used. Lipid extraction methods are time consuming and other approaches like Gerber, creamatocrit, and spectroscopy have been applied for the quantification of lipid in HM. The Gerber method is based on protein precipitation and further separation of fat by centrifugation. A collaborative study (273) performed on crude and homogenized pasteurized cow milk showed that the Gerber method underestimated the amount of fat by 0.02–0.06% compared with the official method of Röse-Gottlieb. A critical point of the method is the accuracy of butyrometers. The supplier should calibrate the butyrometer at delivery and supply a test certificate. The internal surface of the butyrometers must be smooth and free of defects. Recently, spectroscopy techniques have been employed to measure total lipid in HM, and showed conflicting results on precision and accuracy of these techniques (258, 265, 266). The reasons were mainly due to selection of chemical reference methods for the comparison and calibration of spectroscopy instruments. Therefore, the Folch, Bligh, and Dyer and Röse-Gottlieb extraction methods are highly recommended to perform absolute quantification of lipid in human milk (261) and they have to be used for calibrating near- and mid IR spectroscopy equipment.

##### Lactose

Indirect methods, such as colorimetric methods can show poor selectivity. Casadio et al. (265) reported the lack of selectivity of the enzymatic essay to measure lactose in HM, due to lactose overestimation when compared with HPLC measurements. Attempts have been made to quantify lactose by near- and mid IR spectroscopy (258), however neither of the approaches were accurate or precise (261, 266).

Below the main considerations, for successful HM collections and analyses in observational as well as interventional studies, are summarized (see Table 5).

**Table 5:** Recommendations for collecting and handling of HM samples.

**Collection of samples**
Standardize the time of sampling of HM to avoid diurnal variations
Agree on breasts for HM collection
Request mothers to completely empty the breast, ~2 h prior to collection of milk by feeding the infant or manual pumping to minimize the impact on nutrient concentration of interest by residual milk from previous feeding session
Realize single full HM sampling to ensure inclusion of fore-, mid-, and hind milk
Record the volume of milk
Standardize the collection of milk for all study subjects by either electrical pumping or hand pumping to minimize variation due to extraction method
**Handling of samples**
Store milk in smaller aliquots (e.g., 250 μL/aliquot) to avoid freeze-thaw cycles if multiple analyses are anticipated
Store aliquots of milk in tubes at −80°C as quickly as possible and at temperatures as low as possible in field collections until transfer to central laboratory for longer term storage
**Analyses**
Choose appropriate analytical method to characterize and quantify nutrients in HM, with respect to their selectivity, accuracy and precision

## Conclusions and Further Recommendations

To our knowledge, this is the first comprehensive literature review that provides a holistic overview of a broad range of factors affecting HM composition and volume. Several maternal and infant characteristics as well as study methodological aspects were found to impact HM composition and volume. Study procedures should be systematically recorded and/or standardized in individual studies, whenever possible, to facilitate appropriate analysis, and interpretation of data and comparisons across studies. HM lipids and lipophiles were the nutrients found to be most vulnerable to maternal, infant and methodological factors. We therefore recommend standardizing the sampling of the HM, especially for characterization of lipids, lipophiles, and for those nutrients whose variability is yet unknown. Keen attention should be given to the choice of the analytical method to characterize and quantify nutrients in HM, with respect to their selectivity, accuracy and precision.

Our review not only summarizes recommendations for standardization of HM sampling and handling for future research protocols, but also sheds light on the dynamic and highly variable nature of HM. This knowledge is crucial to better understand the controversial impact HM may have on developmental outcomes of infant early life. We also illustrate the agility of HM to adapt to changes in the environment of the mother and evolving nutritional requirements of the infant. There is an apparent need of systematic reviews of existing evidence in the field. It is particularly crucial to better understand those maternal dietary, lifestyle, and environmental exposures that can be modified to significantly impact HM composition and thereby positively impact infants' developmental and health outcomes.

## Author Contributions

ST conceived the idea of the review paper. TS, QZ, FG, and ST wrote sections of the manuscript with critical inputs from DM and VV. All authors read and approved the final version of the manuscript.

### Conflict of Interest

TS, FG, and ST are employed by Nestlé Research. QZ was an employee of Nestlé Research when this work was undertaken, and is currently employed by School of Public Health, Peking University, Beijing, the People's Republic of China. DM has received consultancy payment from Dairy Goat Co-operative (NZ) Ltd. and has given paid lectures for the Merck Sharp & Dohme and Bayer. VV has received funding from Nestlé Research to contribute to the writing of this review while she was employed by INSERM, France.

## Footnotes

^1^2 FL, 2′-fucosyllactose; i, 3′-Fucosyllactose; 3 SL, 3′sialyllactose; AA, Amino acid; BCA, bicinchoninic acid assay; EGF, epidermal growth factor; FM, fat mass; b-FGF, fibroblast growth factor; GDM, gestational diabetes mellitus; GPC, glycerylphosphorylcholine; HM, human milk; HMO, human milk oligosaccharides; IR, infrared; IGF-1, insulin-like growth factor-1; MFG, milk fat globule; NPN, non-protein nitrogen; PCBs, polychlorinated biphenyl; PCDFs, polychlorinated dibenzo-furans; PCDDs, polychlorinated dibenzo-p-dioxins; TN, total nitrogen; TMAO, trimethylamine N-oxide.

## Abbreviations

HM: human milk
PUFA: polyunsaturated fatty acid
LA: linoleic acid
ALA: alpha-linolenic acid
DHA: docosahexaenoic acid
FA: fatty acid
AA: arachidonic acid
EPA: eicosapentaenoic acid
DPA: docosapentaenoic acid
GPC: glycerylphosphorylcholine
TMAO: trimethylamine N-Oxide
BMI: body mass index
Ig: immunoglobulin
EGF: epidermal growth factor
PCBs: polychlorinated biphenyl
PCDDs: polychlorinated dibenzo-p-dioxins
PCDFs: polychlorinated dibenzo-furans
GDM: gestational diabetes mellitus
IL: interleukin
MFG: milk fat globule
HMO: human milk oligosaccharides
b-FGF: fibroblast growth factor
IGF-1: insulin-like growth factor-1
TN: total nitrogen
NPN: non-protein nitrogen
HMA: human milk analyser.
